# mHealth and the management of chronic conditions in rural areas: a note of caution from southern India

**DOI:** 10.1080/13648470.2016.1263824

**Published:** 2017-02-08

**Authors:** Papreen Nahar, Nanda Kishore Kannuri, Sitamma Mikkilineni, G.V.S. Murthy, Peter Phillimore

**Affiliations:** ^a^ Institute of Population Health, University of Manchester,Manchester, UK; ^b^ Indian Institute of Public Health, Hyderabad, India; ^c^ IFHE University, Hyderabad, India; ^d^ School of Geography, Politics and Sociology, Newcastle University, Newcastle upon Tyne, UK

**Keywords:** mHealth, South India, diabetes, depression, medical pluralism, informal providers, RMP

## Abstract

This article examines challenges facing implementation of likely mHealth programmes in rural India. Based on fieldwork in Andhra Pradesh in 2014, and taking as exemplars two chronic medical ‘conditions’ – type 2 diabetes and depression – we look at ways in which people in one rural area currently access medical treatment; we also explore how adults there currently use mobile phones in daily life, to gauge the realistic likelihood of uptake for possible mHealth initiatives. We identify the very different pathways to care for these two medical conditions, and we highlight the importance to the rural population of healthcare outside the formal health system provided by those known as registered medical practitioners (RMP), who despite their title are neither registered nor trained. We also show how limited is the use currently made of very basic mobile phones by the majority of the older adult population in this rural context. Not only may this inhibit mHealth potential in the near future; just as importantly, our data suggest how difficult it may be to identify a clinical partner for patients or their carers for any mHealth application designed to assist the management of chronic ill-health in rural India. Finally, we examine how the promotion of patient ‘self-management’ may not be as readily translated to a country like India as proponents of mHealth might assume.

## Introduction

The term mHealth refers to the delivery of health-related services via mobile communications technology. The potential of mobile phone technology to extend the reach of health care has been attracting increasing global attention and investment, with accompanying claims about its possibilities (Malvey and Slovensky 2014; Mosa, Yoo, and Sheets 2012; Qiang et al. 2011; WHO 2011).[Fn en0001] While initial advances in mHealth are predictably taking place in the wealthiest countries where health systems and media technologies are most developed, much of the advocacy for mHealth concerns its potential in developing countries, where large segments of the population are scarcely served by health services (Arie 2015; Kahn, Yang, and Kahn 2010). However, there is also a growing literature questioning the more enthusiastic claims of mHealth proponents, and calling attention to some of the complexities involved in scaling up from small-scale pilot projects (Chib, Velthoven, and Car 2015; Mechael 2009; Tomlinson et al. 2013). Little of this cautionary literature, however, is grounded in empirical consideration of potential local contexts of use. Our paper contributes to discussion of these complexities, based on qualitative research in rural India. We identify some of the difficulties which will need considering by mHealth advocates and planners in a country known for the complexity of and gaps in its health system.

Our analysis draws on data collected in rural South India (Andhra Pradesh). It stems from the initial phase of an applied multi-disciplinary project whose primary purpose was to design and test a mobile phone application to assist those with chronic health problems.[Fn en0002] Two medical ‘conditions’ were selected for illustrative purposes, both of growing importance in India as elsewhere: type 2 diabetes and depression. This first phase of research was anthropological in scope.[Fn en0003] It entailed an exploration in one rural locality of how diabetes and depression are currently understood, diagnosed and treated, and how mobile phones are currently used. The purpose of this initial stage was to identify some of the challenges involved in developing a practicable mobile phone application to assist and enhance self-management of these two conditions in rural India.

To date, the literature on mHealth applications in rural India has been limited (DeSouza et al. 2014). It has tended to anticipate the potential of the technology to support staff working in primary care settings, rather than to reflect on lessons from implementation (Ajay and Prabhakaran 2011; Ramachandran et al. 2013). Thus, hypothetical benefits in terms of remote clinical support by district-level clinicians, or as an aid to patient ‘adherence’ in the case of chronic conditions such as HIV or diabetes, have been identified by the authors above, along with some indications of anticipated acceptability to patients of using mobile phones for support with their own health care (Bali and Singh 2007). One limitation of this literature is that it makes assumptions about treatment pathways which may bear little relation to actual practice by patients or their families. The present paper therefore introduces considerations which have hitherto largely escaped attention.

Our broad aim is to examine how straightforward it may be to achieve the claimed potential of mHealth in rural India. With regard to patient communication, policy models of mHealth intervention have yet to reckon with the major challenge of how to ‘reach’ the patient. This is all the more challenging where patients bypass or avoid the formal health system (WHO 2011). For instance, when day-to-day treatment needs are partially met outside the formal health system, does this highlight the potential value of mHealth initiatives to fill the gap? Or conversely might it show how complicated the prospects for mHealth initiatives are likely to be? In these contexts, knowledge of the pathways that patients follow to obtain treatment is a crucial first step towards thinking about what kind of mHealth applications might work. Our data lead us to an aspect of medical pluralism in India which may well complicate potential mHealth interventions, and we highlight particular difficulties that may be faced when planning mHealth applications to support those with depression. Crucial here is the importance in rural areas of an informal practitioner known as RMP (defined below), whose significance in relation to mHealth interventions we discuss at some length. A further consideration, to which we return in the Discussion, concerns a tension between assumptions of global mHealth advocacy concerning patient ‘self-management’, and local notions of the self and ‘self-management’ in an Indian health care context. There has to date been no attempt to consider the implications of contrasting cultural notions of the self in this context, and we seek to identify some of the issues raised by a trans-national policy discourse of ‘self-management’.

There are no easy answers to the questions we pose. Our main purpose is to highlight dilemmas which, we judge, may face mHealth initiatives in rural settings in developing countries. First, we present evidence on access to treatment for diabetes. Second, we examine challenges individuals face in obtaining treatment for depression. In each case, we keep in mind the possible obstacles to providing viable mHealth support. This leads to an overview of current mobile phone use in the research setting as a benchmark against which to reflect on challenges for mHealth policy initiatives. The Discussion pulls together the main implications of our analysis, particularly in relation to communication between clinic and patient, and the potentially vexed issue of ‘self-management’.

## Background

Diabetes and depression might seem an unexpected pairing as exemplar chronic ‘conditions’. They were selected for three main reasons. First, the prevalence of both is growing worldwide, and each is viewed as an increasingly urgent health priority. Second, in rural settings access to health care is recognized to be an even greater concern than in urban settings, while the chronic nature of diabetes and typically depression was seen to pose particular health care difficulties. And third, it was precisely the marked differences between the social and somatic experiences of diabetes and depression – including the pronounced stigma often attached to the latter – which proposed them both as a valuable contrast, not least to explore what the notion of ‘self-management’ might mean for those living with each.

Of the two, it is more straightforward to summarize the epidemiological and demographic background for diabetes. India now has a massive problem with diabetes, even if prevalence remains lower than in parts of the Middle East (Diamond 2011; Mohan et al. 2008; Ramachandran, Ma, and Snehalatha 2010). While it was initially seen as a disease of the affluent and sedentary, diabetes now afflicts many sections of the Indian population. Prevalence is still reported to be considerably higher in urban settings; however, rural areas are now no stranger to diabetes (Mohan et al. 2008). Popular explanatory models in India broadly accord with biomedical explanations, and the term ‘diabetes’ has itself become widely familiar. While diabetes in middle age or older does not seem to attract social stigma, it may be a different matter at younger ages, notably for young women with type 1 diabetes (Kalra, Kalra, and Kumar 2009). That said, type 2 diabetes is a relatively straightforward medical ‘condition’ to investigate empirically, as it is neither socially hidden in middle age nor epistemologically elusive, even if there remains uncertainty over the accuracy of epidemiological estimates (though see Ferzacca 2012 for a global anthropological review).

Unlike diabetes, the term depression may well correspond poorly with vernacular idioms across India, and thus its use by medical staff (or researchers) may have limited meaning or salience to those who are affected (Chowdhury, Chakraborty, and Weiss 2001; Jain and Jadhav 2009; Raguram et al. 2001). Moreover, the stigma surrounding any kind of mental ill-health helps push depression out of sight, especially in rural areas (Jadhav et al. 2007). And because mental illness, including depression, has largely been suffered beyond the reach of medical surveillance, epidemiological efforts to estimate prevalence range widely and remain tentative (Grover, Dutt, and Avasthi 2010; Poongothai et al. 2009). Selecting depression for the present study, therefore, posed challenges going well beyond those posed by selecting diabetes.

The third component of this research concerned mobile phone use. Qualitative research on the place and significance of mobile phones in people's lives in India has grown rapidly. Most influential has been Jeffrey and Doron's (2013) recent study, combining detailed ethnography from Uttar Pradesh and Kerala with a pan-Indian overview. They are not alone in representing the mobile phone as an exemplary and transformative ‘technology of the self’ (see Horst and Miller 2006). However, challenging any automatic equation of mobile technology with individualization and the self, Sreekumar takes the example of fishing livelihoods in Kerala. He posits ‘the collectivist logic in a community's appropriation of new technologies’ (2011, 172), when examining the importance of mobile phones for two critical activities – safety at sea and fish selling. And he argues, against the grain of most writing, for recognizing those contexts where the mobile is better regarded as a ‘collectivist machine’ (2011, 178). We return later to these debates concerning the self, as they are germane to modern discourses of self-management.

## Setting

Our research took place in rural Andhra Pradesh, in a district, Guntur, which forms part of coastal Andhra. Guntur had a population of 4.9 million in 2011, with two thirds classed as rural. This is an area with a long history of commercialized agriculture (Upadhya 1997). Extensive irrigation a century ago provided a foundation for growing wealth, one of whose consequences has been relatively high levels of literacy for many decades (Upadhya 1997). This, however, is not to minimize the pronounced inequalities which persist in the countryside, where land remains concentrated in the hands of a few dominant castes.

Guntur was selected as a district because it was judged that the relatively high levels of literacy and education would be helpful to a study with a primary ‘proof-of-concept’ purpose. The main research setting comprised two neighbouring villages 30 kilometres south of the city of Guntur. Thus, the setting was rural but not remote. Both villages had sizeable proportions of Hindus, Muslims and Christians, like most villages in the area. Nagulapadu had a population at the 2011 Census of 5400 and was majority Hindu, while Kommuru, had a population of 6600, and was majority Muslim. Overall, households in Nagulapadu were more affluent than those in Kommuru. In both villages, the Christian minority identified themselves as Dalit.[Fn en0004] They comprised the poorest section of the population. Each village had a primary health sub-centre. While these two villages were the focus of our fieldwork, we also conducted research in two private psychiatric clinics in the city of Guntur, for reasons explained below: one an independent clinic, the other located within a teaching hospital.

## Methodology

Data collection in the two villages combined interviews with group discussions, ethnographic conversations and observation. Semi-structured interviews were conducted with 21 individuals suffering from diabetes (10 men, 11 women). We identified prospective diabetic interviewees through the local knowledge of Auxiliary Nurse Midwives (ANM) and Accredited Social Health Activists (ASHA), based in the two primary health sub-centres.[Fn en0005] Semi-structured but more extended interviews also took place with a variety of health workers serving these villages who had been identified as local key informants (11 in all): one ANM, one ASHA, one PHC doctor, one PHC laboratory technician, one homeopathic doctor, one pharmacist, four registered medical practitioners (RMP), and one herbalist who was described as a ‘traditional healer’. Five organized group discussions (with 4--10 in each group) were conducted with villagers who did not have diabetes; while opportunistic fieldwork conversations also took place with a selection of other figures, including several who were locally prominent – for example, village council (*panchayat*) office holders, shopkeepers, and a Hindu priest – to help gauge wider community knowledge about the two selected ‘conditions’ and mobile phone use. These interviews and conversations were supplemented with observations of both Government and private clinics’ practice, and RMP consultations. Interviews were also conducted with two mobile phone sellers and one mobile phone repairer.

Although we enquired about local understandings of depression, we did not interview those diagnosed as suffering from depression in the two villages. Instead, we interviewed a sample of 21 individuals (13 men, 8 women) from rural areas who travelled to either of two clinics in the city of Guntur for psychiatric outpatient consultations (17 attended one clinic, four the other). Prospective interviewees were selected by senior clinic staff, and interviews took place within the clinic. We also interviewed two psychiatrists (one from each clinic) and one clinic manager. We recognize that our two samples of interviewees were based on different recruitment criteria. Such a difference in approach reflected the considerable stigma surrounding mental illness (Jadhav et al. 2007; Kannuri 2015). This we had anticipated; but the reticence around the topic in the course of initial enquiries in the study villages also confirmed our view that it would be difficult and intrusive to seek interviewees there. The decision to approach those with depression through the clinic where they were being treated was therefore judged a necessity in the circumstances.

The team designing the study and supervising data collection comprised three social anthropologists (PN, NKK, PP) and one psychologist (SM), working under the auspices of a leading public health research institute and its director (GVSM). Interviews with patients were undertaken by two field assistants specially trained for the job. Interviews with key informants and group discussions involved at least one of the authors (PN, NKK and SM, the last two of whom were Telugu speakers). These three also guided the ethnographic observations. Interviews with patients and health staff were audio-recorded with consent (with one refusal), and subsequently transcribed and translated from Telugu into English. Fieldwork took place over six months, January--June 2014.

Criteria for selecting interviewees with diabetes or depression were established in advance on the basis of clinical advice. We kept in mind that no individual should be too unwell to be able to use a mobile phone. For those with type 2 diabetes, we were advised to select the 30--59 age range, and excluded those considered to have serious diabetes-related complications. For those with depression, we were advised to adopt a wider age range (18--64). We interviewed those who were available while attending for clinical consultations, stipulating that they should have been diagnosed at least 12 months previously. In practice, nearly all were in their thirties. We excluded those with acute or complex symptoms, as well as those who were considered to be potentially suicidal, violent, or known to have a drug or alcohol dependency. We also excluded women with post-natal depression. Finally, we excluded those who had been diagnosed with both diabetes and depression, though we recognize that co-morbidity is an important category in itself (Mendenhall 2015).

Of the 42 participant ‘patients’, 10 (9 women, 1 man) had no schooling, 8 (6 women, 2 men) had been educated to primary and 9 (3 women, 6 men) to secondary level. A further 7 (all men) had higher secondary education, while another 7 (2 women, 5 men) had attained a Bachelor's Degree. The gendered pattern of education is thus pronounced.

## Access to treatment

### Individuals diagnosed with diabetes

How is diabetes commonly diagnosed, treated and managed in rural Guntur? Our data suggest that those with diabetes nearly always seek treatment that is biomedical (or allopathic, to use the locally common label). ‘Diabetes’ itself is a familiar term, although colloquial usage is typically ‘sugar’ or ‘sugar disease’.[Fn en0006] Type 2 Diabetes appears to carry little or no stigma locally among the age group we studied. Indeed, it is seen as a mark of modest affluence (echoed in remarks that Dalits rarely got diabetes). People with the disease and family members alike spoke readily about it. Diabetes was regarded as a ‘modern’ disease, thought of as being caused by ‘modern’ ways of living, and only effectively treated with ‘modern’ (allopathic, sometimes ‘English’) medicine. One remark was typical of several: ‘In my opinion there is no medicine for this sugar disease. There is only one chance to get the sugar reduced and that is by maintaining proper English medicines’.

Yet the initial treatment is unlikely to be sought in a recognized clinic, whether government-run or in the private sector. It is much more likely that the first port of call in Nagulapadu or Kommuru would be an informal practitioner known as a RMP. Most families know several local RMPs. RMPs have become used to treating diabetes, and those we spoke with indicated that the disease was becoming increasingly common in the area.

Who are RMPs? RMPs are informal medical practitioners, generally but not invariably presenting themselves as practising allopathy. All were men in our area of study. They play a crucial role in rural areas. They practise with no formal medical qualification, and typically flourish where the formal health sector – both government and private – is absent or largely avoided (except for maternal and child health services). There is a small but growing literature on RMPs or their equivalents (not all writers use the term RMP) by social scientists from different parts of India: Ecks and Basu (2009, 2014), Gautham et al. (2014), George and Iyer (2013), Lakshman and Nichter (2000), May, Roth, and Panda (2014) and Pinto (2004). Some writers identify the ‘R’ in RMP as standing for Rural, but in Guntur and indeed more widely in the south it was said to denote ‘Registered’: an irony, as RMPs have historically never been registered and most remain unregistered.[Fn en0007]

The main benefit of RMPs for their clients is that they are local, available and familiar. They charge affordable amounts, typically around Rs. 50 per treatment. Moreover, they are easily accessible for follow-up treatment, a crucial consideration with a chronic condition like diabetes. With these advantages, a good many built considerable trust among their clientele, despite their limited medical skills and rudimentary facilities.[Fn en0008] Some people referred to their RMP as a doctor (an ‘RMP doctor’, as opposed to the ‘big doctor’). A more common alternative name that patients used was ‘compounder’, for a good proportion of RMPs first acquired their medical knowledge when working as an assistant (compounder) to a qualified doctor.

The formal health system did not recognize RMPs at all. Yet several factors counteract such non-recognition. For instance, there have been arguments in government policy-making that RMPs should have a role in implementing Ministry of Health and Family Welfare programmes, as part of a national effort to enhance healthcare in rural areas (May, Roth, and Panda 2014; Rao 2005). More than most states in India, Andhra Pradesh (before its division in 2014 into two states) had started to offer certification and training, partly in response to a long campaign by RMPs themselves. This thereby allows RMPs to claim that they are indeed ‘registered’. Thus, there have been limited top-down and bottom-up pressures for greater acknowledgment of RMPs, though such pressures invariably come up against the institutional resistance of biomedical elites.[Fn en0009]

More significant than these limited policy initiatives is the evidence of routine practice. While the RMPs in our study had virtually no connection with state medicine, we found well-established links between reputable private clinics in Guntur and village RMPs who refer their patients for more expert assessment and treatment. RMPs receive commissions for their referrals, making this moderately lucrative for them. Some RMPs said that they also sought informal medical advice and information from staff at these private clinics to assist their own practice. In short, most RMPs form part of an informal referral network assisting private hospitals or clinics in cities to reach rural clients. Yet private clinics in the city charge their clientele considerable sums and individuals use them sparingly, preferring the services of the local RMP wherever possible, as the comment below suggests:

I have visited two diabetes specialists in the past. [Now] we are unable to pay [Rs.] 2--3000 for tests and everything. I take help from the RMP now. I don't go to his clinic. If I don't feel well I phone him and he comes to my house and gives [me] medicine.

What do these findings mean for the feasibility of mHealth applications? We did not set out to highlight the role and importance of the RMP. That emerged from fieldwork, as a consequence of our enquiries among patients and their families about preferred options for diagnosis and treatment of diabetes. It became apparent that state primary care plays only a small part in the treatment of diabetes: instead the entry point into treatment is much more commonly via the RMP. Their ubiquitous presence and their ready connections with private urban clinics amounts to a treatment pathway that future mHealth planners are likely to have to reckon with. We defer to the Discussion further consideration of what this might mean for mHealth. But such findings provoke various questions. For instance, might mHealth initiatives build on the pivotal position of RMPs, providing inter alia a path to their greater recognition? Or alternatively, might mHealth just as readily offer potential technological solutions that eventually bypass RMPs, in the process marginalizing them further?

### Individuals diagnosed with depression

The steps to treatment in a psychiatric clinic in Guntur reveal marked contrasts with the pathways to treatment we have outlined for those with diabetes. The very term ‘depression’ is deeply problematic. Moreover, the role of the RMP, so central in village treatment of diabetes, is more marginal for those suffering depression. Partly as a consequence, the route to treatment in a psychiatric clinic was often circuitous. Some individuals did indeed seek help initially from their local RMP: but they typically presented with physical symptoms or sleeplessness, rarely admitting to any kind of mental distress. In such cases, an individual was likely to come to a specialist psychiatric clinic after referral by another private clinic in the city. Nonetheless, there were occasions when psychiatric clinics evidently did take patients referred directly to them by RMPs, and reluctantly admitted to doing so. Additional suitably anonymous pathways to psychiatric clinics included advertisements in local media (which have become increasingly common in recent years); while auto-rickshaw or taxi drivers may also bring an individual to the clinic after a new arrival at the bus station had sought advice on where to find help. Referrals by religious institutions – temples, mosques or churches – appeared to be rare. Yet patients themselves had commonly sought religious or ritual forms of healing beforehand, an evident contrast with treatment-seeking for diabetes.

These circuitous routes to treatment were a predictable consequence of deep fear of public knowledge of mental illness, which usually led individuals (or family members) to avoid consultation with any local practitioner, including an RMP. For the distress of mental health problems was compounded by a fear of gossip and exclusion, feeding a desire to seek treatment with the utmost discretion. As one interviewee said:

I don't intend to tell anyone about my depression, not even my close friends. Even if I tell them, they won't understand. They will keep asking me the reason for it. Then I must tell them about personal matters and they would make fun of it. People are not broad-minded.

Most of the individuals we interviewed had been referred for psychiatric treatment by other private clinics in the city after first presenting with somatic problems. Almost all reached the clinic having had no prior diagnosis for depression. In every case, moreover, clinical symptoms of depression were relatively severe. Even though we had sought to exclude the most severe or complex cases, invariably symptoms had to be severe and disabling before even being considered worthy of medical treatment by the individual (or family). There was thus no instance of ‘mild’ depression in our sample, as judged by the clinic staff. While both clinics formally insisted that outpatients be accompanied by a relative, there were exceptions to this rule. Some individuals felt that they had to conceal the problem from everyone in their household and family, and would be unwilling to let anyone know.

Within the growing anthropological literature on mental health in India, two broad lines of exploration have been prominent. Various writers (Ecks 2005; Ecks and Basu 2009, 2014; Jain and Jadhav 2009) have examined some of the profound cultural and economic complexities of the heavy reliance on pharmacological treatments, including in rural areas and informal practice. Others have explored what Lang and Jansen (2013) call the ‘appropriation of depression’ by biomedical frames of understanding (see also Halliburton 2005; Quack 2012). In line with this latter view, those we interviewed had learned, through the clinic, to think of their symptoms as signs of something known as ‘depression’, even though this term had little local salience. Nor did it translate straightforwardly into any Telugu idiom, as diabetes did. In practice, the commonest approximations were English words – ‘stress’ ‘pressure’ and above all ‘tension’ (cf. Halliburton 2005).[Fn en0010] ‘Tension’ indeed evoked wide semantic associations, for it was readily used to describe the character of modern living and was acknowledged to be a growing local health problem.[Fn en0011]

## Mobile phones and their current use

To consider the feasibility of mHealth applications in rural settings, it is important to have a picture of current habits of mobile phone use. Nearly all the individuals we interviewed, with diabetes or depression, possessed their own mobile phone (34); only four of our sample (all women) had no access to a mobile. These were virtually all cheap 1G or 2G mobiles, with only two possessing smart phones. According to the mobile phone sellers we met, most mobiles cost Rs.2000--4000 (£20--40), with 4--8 GB memory.[Fn en0012] Households typically had two or three mobile phones, with men and those of student age (especially the latter) dominating usage. Second-hand mobiles were available from local sources (including mobile repairers), but most users purchased new mobiles, while the old phone got passed on within the family. A familiar discourse was affirmed in our findings: of adult men using phones for business while women used them to keep in touch with family members (Jeffrey and Doron 2013). However, a number of women said they needed help making calls and were more familiar with receiving, while no men admitted to needing such assistance. Similarly, men claimed to text more than women, though neither texted much. None of our interviewees said they used apps on their phone, though some knew that their children did. Because providers offer different cheap-rate packages, there was some use of dual-sim cards in the area. However, mobile phone sellers reported that it was mainly young people who did so, and this assessment was borne out by our interviewees and others we spoke with.

None of our interviewees had a contract, and pre-paid use was the norm. Typically, individuals topped up with Rs.200--250 per week, though in poorer households that sum might be halved. Within both villages, shops sold top-up cards for very small amounts (Rs.10). Overall, while some households spent little more than Rs.10 per month, others reckoned to spend around Rs.1000. Differences in use and wealth were also apparent in approaches to charging mobiles, ranging from those purchasing branded chargers, to the poorest paying to charge in shops. Landowners especially highlighted the value of having phones with batteries which would last a full day, given the unreliability of electricity supply during the rainy season.

But did mobile phones have any current uses for health care purposes? Those we interviewed in the two villages certainly used their mobiles to call their RMP. A few spoke of calling the private clinic they attended in Guntur, but likewise simply to make or check appointments. There is nothing in our data to suggest that mobiles were used for any additional clinical purpose. Might families, however, use their mobiles to support each other's care? To some degree this occurred, though few were forthcoming about its daily utility in this regard. One woman with diabetes, who said she had little interest in her mobile, nevertheless added:

Generally when I am alone, I neglect [myself]. I don't cook. They [family] know. That is why they call and tell me to cook and eat properly. They remind me.

Another woman with diabetes saw the mobile pressed on her by her absent children as redundant:

My children sent me a phone from abroad. I have no interest in using it. My children always tell me to use one. But I don't. My husband and children are enough for me [to help remind her].

In relation to those with depression, while a patient could in theory contact their psychiatric clinic, in practice it was rare. None of those we interviewed with depression spoke of using their mobile phone to call their clinic; it was always the other way around, with clinics providing appointment reminders or when required a ‘lifeline’ service. As we were reminded by clinic staff, individuals suffering from depression are perhaps among the least likely people to turn to their mobiles in the first place.

In sum, therefore, widespread ownership of basic 1G or 2G products does not (yet) translate into a pattern of habitual use which suggests easy take-up of mHealth applications by patients themselves. At present, moreover, basic phones, seasonally erratic electricity-supply, or short battery life, each constrain habits of use, with potential implications for the reliability of access for mHealth communication in the near future.

## Discussion: lessons and implications

mHealth applications are anticipated as a potential response to pressing problems of public health surveillance and future health service provision. Access to services in rural areas is one such problem on a global scale; and diabetes and depression provide exemplar medical conditions through which to explore some of the challenges involved. A number of mHealth developments in rural health care have envisaged enhancing communication between different tiers within a health system (notably to provide remote clinical support to local staff). That may well be a feasible aim. Other options more ambitiously envisage ways to improve communication between health providers and patients. The latter – the focus of our project – poses greater challenges. Our evidence leads us to raise a note of caution, suggesting that some of the more ambitious hopes for mHealth applications may prove hard to realize in parts of rural India for the foreseeable future (cf. DeSouza et al. 2014). There are several factors to tease out here arising from our data. Three seem particularly salient, each concerning the actions and agency of the individual patient (or family members). First, there is the tendency we noted for many patients with diabetes to avoid the government sector, or indeed the formal health sector as a whole. The role of the RMP is central to such choices. Second, we consider lessons from the particular difficulties entailed in seeking and sustaining treatment for ‘depression’. Finally, we reflect on the notion of the patient as an actor in managing their own health care, expressed in terms of the benefits of patient ‘self-management’.

Our study highlights a critical issue – that of communication between clinic and patient. Proponents of mHealth may acknowledge that it might be problematic to ‘reach’ the patient; but they assume it is likely to be straightforward to identify the appropriate clinical end of the communication. Our evidence leads us to question that assumption. The local popularity of the RMP is emblematic of the task facing mHealth planners in this regard. In Nagulapadu and Kommuru, the popularity of the RMP as a medical practitioner coexists with the unpopularity of government-run services. The latter at present therefore seems a scarcely credible option for mHealth communication with diabetes patients. More broadly, the formal health sector – state-run or private – simply does not have the reach in rural areas that it purports to have (cf. Pinto 2004). Thus, as we asked earlier, might rural mHealth initiatives therefore build on the pivotal position held by RMPs? That likewise seems implausible at present. For many, this may reaffirm the urgent need for mHealth to help fill the gaps in the formal health system. Yet our study suggests some of the conundrums facing efforts to translate a defined need into a practicable policy solution. Thus, we return to our initial question: who might provide the clinical communication to sustain remote monitoring and support of patients with chronic conditions like diabetes via mobile applications?

The potential problems for mHealth applications to support those diagnosed with depression are different. Such a diagnosis is itself only likely to come from a recognized psychiatric clinic, whether government-run or private. In this context, psychiatric clinics may appear well-placed to initiate mHealth support, and indeed in practical terms they have the infrastructure and access to make use of such developments. Yet how responsive is the potential patient user likely to be? Where enormous effort goes into concealing such a stigmatizing illness and identity, especially in rural areas, it seems uncertain at best how easily the intended users will feel it may be ‘safe’ for them to use a novel application which may prove difficult to use discreetly. Nothing in our data encourages the judgement that the kind of individual we interviewed would readily use their mobile phone anyway: possession of a mobile did not necessarily mean a habit of usage. Thus, the case of depression highlights some of the difficulties – not necessarily insuperable – facing mHealth initiatives for patient support.

This leads us to our final point. Certain mHealth applications to support communication between health providers and their clientele posit a hypothetical patient who is not merely a passive recipient of information from their clinic but is instead a more active participant in monitoring their own health. Here, we reflect on some of the implications of this expectation. There is little sociological literature exploring ‘self-management’ in healthcare for those with chronic conditions in rural India (though see Staples 2004). Both the assertion of medical authority and wider cultural inequalities mean that aspirations to patient empowerment go against the grain, above all in rural areas of India. But there are also additional complexities to implementation of mHealth initiatives which assume self-management. One of these concerns the premise of the adult individual acting as an independent agent within the family setting. This is relevant not only to health decisions generally but also to mobile phone use. While several authors see the mobile phone as a ‘technology of the self’ (as its manufacturers do), Sreekumar's work (2011) on Keralan fishing communities reminds us that the mobile phone may serve a more collectivist purpose (see also Doron 2012). This is an important counter to easy assumptions about individualism in health decisions or mobile phone use.

The very precept of ‘self-management’ of one's own health owes much to philosophical assumptions about the self and individuality: the notional figure of the self-managing individual fits well with popular western notions of self-actualization; but it is equally part of the rhetoric of neoliberal reform in finance and governance advocated by international bodies for health systems around the world (see Jeffery and Jeffery 2008). Any mHealth application to assist patient self-management encodes crucial assumptions from this global discourse. And it begs the question of how much purchase such tacit assumptions may gain when translated into a rural Indian context, with distinctive understandings of the self and the individual. A large ethnographic literature from India is relevant here. One contrast made in this literature distinguishes between more ‘autonomous’ and more ‘relational’ notions of the self, the latter said to better typify the self in relation to the wider family.[Fn en0013] However, various writers have also warned against overdrawing this distinction in India (indicatively, Busby 1997; Lamb 1997). Lamb in particular suggests – from her ethnography in rural Bengal – that the balance between the ‘relational’ and the ‘autonomous’ anyway tends to shift over the life-course in a gendered way. Lamb's emphasis on gender and the life course is helpful in considering the distinctive experiences of those in this study with diabetes or depression, and the potential for mHealth support. Where her analysis cannot assist, however, is in charting how one generational cohort may differ from the next – how the experience and conception of ‘self’ of those who are now middle-aged will differ from their children's future experiential sense of self. Yet this will be vital to understanding potential habits of use of the mobile phone in mHealth developments over the next generation.

This was a study in one rural area of India, and itself one strand in a larger empirical project scoping the potential for mHealth applications to assist with the management of chronic ill-health. While we recognize the need to be cautious in generalization, it does nevertheless point to some of the social and systemic challenges (as opposed to those that are simply technical) that may be faced in rural India in what is likely to be an intensifying push by corporations and governments (Qiang et al 2011; WHO 2011) towards mHealth applications as a response to health system limitations.

## Notes

1. The opening words of the WHO report cited here state (2011, 1): ‘The use of mobile and wireless technologies to support the achievement of health objectives (mHealth) has the potential to transform the face of health service delivery across the globe’.

2. This was designed as ‘proof-of-concept’ research, possibly an unfamiliar and certainly an unsympathetic turn of phrase for an anthropological readership. It referred to the project aim of developing and demonstrating a mobile phone application which met the multiple challenges in India of being (1) technically feasible in rural areas (where connectivity could be especially problematic), (2) a beneficial supplement to treatment or care, (3) straightforward and convenient to use and (4) socially acceptable for its users. Apart from the issue of technical feasibility, all are matters where anthropologists and other social scientists have a distinct contribution to make.

3. Relatively self-contained, this first phase had to fit within and engage with the wider project design and aims. The anthropological contribution was thus particularly to guide and inform colleagues in public health and human–computer interaction.

4. The terms Dalit and Scheduled Caste refer to the ‘lowest’ castes: the former a term of self-description widely used in the media, the latter a government category also in common use. Many Christian groups are among Guntur's elite. That is far from the case in these two villages.

5. Very few of our interviewees with diabetes came from Christian/Dalit households. There may have been some conscious exclusion here. Yet several people also suggested that diabetes was still rare among Dalits, local explanation emphasizing the physically active nature of the lives they often led.

6. Local Telugu names existed (*madhumeham*, or *chakkeravyadi*, a literal translation of ‘sugar disease’), but these had been largely supplanted.

7. George and Iyer (2013) offer useful historical background for this definitional issue. See also our own analysis of RMPs (Nahar et al. forthcoming).

8. While Gautham et al. (2014), like us, emphasize their practical importance in rural healthcare, Jeffery and Jeffery (2008, 2010) highlight the damaging role RMPs may play in obstetric care in rural Uttar Pradesh.

9. The Andhra Pradesh Chapter of the Indian Medical Association is unequivocal in its rejection of RMPs in its campaign against ‘illegal medical practice’ and ‘quackery’: http://www.imaapstate.com/downloads/32%20anti%20quackery.pdf

10. On occasions, this was expressed in Telugu as *manasikandolana* or *andolana*, conveying the sense of ‘disturbance’ or ‘agitation’ of the mind.

11. The insecurity of agriculture, for example, was regarded as a major source of ‘tension’. This was one context in which diabetes and depression became linked within larger stories of contemporary farming insecurity. Fieldwork conversations about diabetes and food led to reflection on agriculture, which in turn led to the topic of ‘tension’, and the toll this took of people's mental well-being (cf. Vasavi 1994) on ‘hybrid times’).

12. The pivotal role of the mobile phone seller in a Mumbai neighbourhood is illuminated in Rangaswamy and Nair (2010).

13. India was in fact an origin point of extended debate in anthropology a generation ago about concepts of the self and person (cf. Marriott and Inden 1977).
